# Herniary Protrusion of the Stomach

**Published:** 1859-09

**Authors:** C. H. Spilman

**Affiliations:** Harrodsburg, Kentucky


ART. VI.—*Herniary Protrusion of the Stomach into the Cavity of the Chest.* By C. H. SPILMAN, M.D., of Harrodsburg, Kentucky.

JOHN BULL, a free man of color, on the night of the 22d of May, 1857, was stabbed in the left side; the knife entering between the seventh and eighth ribs into the cavity of the chest, passing through the diaphragm into the abdomen, and making an incision in the diaphragm of about two and a half inches in length.

Twenty minutes after the wound was inflicted, I found him much exhausted from loss of blood. Immediately afterwards my friends Drs. Moore and Jones arrived. A portion of the omentum, three inches in length and two in width, protruded from the wound. Finding its connection very slight, and its return *in situ* impracticable, we removed it, applied some loose dressings, administered fifteen grains of calomel and one grain of ipecac., and left him for the night, with the wound dependent.

23d, A.M. Pulse 106; restless and feverish; irritable stomach; bowels not moved; abstracted twenty-four ounces of blood; directed an infusion of salts and senna through the day; enema repeated at intervals of two hours. Two or three pretty copious alvine evacuations in the evening. More quiet, and spent the night in tolerable comfort.

24th, eight o'clock A.M. Pulse 106; some thirst; stomach somewhat irritable; yet the symptoms, taken in the aggregate, rather promising.

Six o'clock P.M. Pulse 110, and tense; sick stomach, with occasional vomiting; pain in the epigastric region; no tenderness on pressure, nor abdominal tumefaction; on the contrary, rather a sunken condition of the abdomen; surface but little above the natural temperature, and harsh and dry. Drew off a few ounces of blood, and gave a full anodyne.

The three succeeding days but little variation in the symptoms. Complained but little of sick stomach; its contents were continuously ejected, not by any active expulsive effort, but by simple regurgitation.

On the sixth day (28th) the tendency was manifestly downward. Pulse 140; expression anxious; thirst insatiable; features sharp; bowels immovably constipated. We were exceedingly puzzled at witnessing these fearfully ominous symptoms, which pointed unmistakably to serious lesion of the abdominal viscera, with the entire absence of tenderness and tumefaction, its invariable accompaniments.

From this period to the day of his death, which occurred on the second of June, every effort to procure an action from the bowels proved unavailing. The stomach received food, water, and medicine, but seemed only a passive receptacle, and nothing passing beyond, and being kept in a constant state of repletion, the regurgitation seemed simply an overflow of that organ.

*Autopsy.*—The sternum elevated by a section of the cartilages as high as the first rib, a longitudinal incision through the parietes of the abdomen, extending as low as the umbilicus, and the removal, by the saw, of the anterior half of the fourth, fifth, sixth, seventh, and eighth ribs on the left side, exhibited a spectacle which fully accounted for the train of morbid phenomena, previously so perplexing and inexplicable. The stomach was found to be greatly distended, and lying in the cavity of the chest, in immediate contact with the apex of the heart and left lung. It had passed by a herniary protrusion through the small incision in the diaphragm, imposing a stricture upon the orifices, and complete occlusion of the pylorus.

The entire organ embraced within the stricture, distinctly marked and limited by its site, was of a dark mahogany color, indicating vascular as well as alimentary obstruction, and presenting all the characteristics of protruded bowel as in cases of strangulated hernia. It contained a large quantity of greenish-black fluid, almost inodorous, suspending, in an unchanged state, portions of the various articles of food that had been taken since the wound was inflicted.

Finding here sufficient to account for the death of our patient, and that anomalous combination of symptoms which marked the case, we prosecuted the examination no further.

The case suggests some curious questions: and

1st. Did the displacement occur at the time the injury was received, or at some subsequent period?

2d. By what internal force could an organ, the size of the stomach, have passed through such a small aperture?

3d. Whether, with a certain knowledge of the dislocation at the time, its reduction by surgical interference was practicable?

These are questions to which we have not, as yet, been able to find a satisfactory answer.

